# Use of 24-Hour Recalls to Assess Adherence to Cancer Specific Dietary Guidelines: Experiences from the Advancing Survivorship Cancer Outcomes Trial (ASCOT)

**DOI:** 10.1080/01635581.2025.2514783

**Published:** 2025-06-06

**Authors:** Victoria Ireland, Helen Croker, Sara Esser, Phillippa Lally, Rebecca J Beeken, Abigail Fisher, Rana Conway

**Affiliations:** a Research Department of Behavioural Science and Health, University College London, London, UK; b World Cancer Research Fund International, London, UK; c School of Psychology, University of Surrey, Guildford, UK; d Leeds Institute of Health Sciences, University of Leeds, Leeds, UK

## Abstract

People living with and beyond cancer (LWBC) are advised to follow World Cancer Research Fund (WCRF) dietary guidelines. However, there is no established methodology to assess adherence. This study aimed to: (i) develop methodology to process dietary recalls into a format comparable to WCRF guidelines and (ii) evaluate the impact of additional data processing on estimates of dietary intake, in people LWBC. Advancing Survivorship Cancer Outcomes Trial (ASCOT) participants completed two 24-h dietary recalls at four timepoints using myfood24. Five WCRF recommendations (limiting consumption of energy dense foods, red meat and processed meat, and increasing consumption of fruit and vegetables and wholegrains and pulses) were operationalized (e.g. ≤ 500 g red meat per week). Quality control checks indicated the need for additional processing, including changing portion sizes and choosing alternative items from myfood24 to improve accuracy. Compared to myfood24 output, the processed dietary data indicated lower intake of fruit and vegetables, and higher intake of NSP and AOAC fiber (all ps < 0.001). Developing methodology to allow assessment of 24-h dietary recall data against WCRF guidelines was possible and necessary but resource intensive. Additional data processing impacted estimates of the key foods and nutrients consumed by trial participants in a meaningful way.

## Introduction

Through earlier diagnosis and improvements in treatments, more people are surviving cancer, leading to a larger population in the UK living with and beyond cancer (LWBC) (1,2). According to the World Cancer Research Fund (WCRF), people LWBC should adhere to the WCRF’s lifestyle recommendations for cancer prevention if they are able to (3). To reduce diet-related risk, the WCRF advises individuals LWBC to limit consumption of red and processed meat, processed foods high in fat, sugar and alcohol; and increase consumption of fruit, vegetables, wholegrains and beans (3). Many adults in the UK fail to meet national dietary guidelines and consume more free sugar and saturated fat and less fruit, vegetables and fiber than recommended and there is little evidence that a cancer diagnosis motivates health protective changes (4–9).

The WCRF has identified a need for individuals LWBC to receive guidance on nutrition, but it is unclear how to provide behavior change support that is simple yet effective (3). The process of developing and evaluating interventions to support dietary improvement inevitably requires an assessment of dietary intake in relation to WCRF guidelines. The use of 24-h dietary recalls is now the most common method of collecting detailed dietary intake data, as participant burden is relatively low compared to diet diaries. When recalls are self-administered *via* online systems this also reduces researcher burden and increases cost effectiveness, although issues with data quality remain, for example due to problems with memory, portion estimation, and social desirability bias (10–15). As WCRF guidelines extend beyond nutrients (e.g. fat) to foods (e.g. red meat), depending on the data output from online recall software, additional processing may be required before dietary data can be used to assess guideline adherence. For example, if the output does not include “red meat,” assessing adherence to the recommendation to limit consumption becomes complex. Other considerations include whether dietary variables assessed by the chosen software are consistent with WCRF recommendations, for example, whether fruit juice is counted as “fruit.” The number of items in a database with missing nutrient composition data also needs to be considered. As a full nutrient profile is generated irrespective of human error or software problems, it is essential that research studies include quality control checks to ensure data integrity (16). In practice, a pragmatic approach may be required to balance a desire for accuracy with available resource for checks, taking into account the volume of data being collected.

The Advancing Survivorship Cancer Outcomes Trial (ASCOT) is a brief intervention using habit-based advice to improve people LWBC’s adherence to WCRF guidelines for diet, physical activity, alcohol and smoking (17). A randomized controlled trial was conducted to determine the effectiveness of the ASCOT intervention, which necessitated addressing the challenges set out above. Developing and performing additional data processing procedures to assess adherence to WCRF guidance presented a challenge which would be resource intensive if implemented at each stage of data collection. As it was unclear whether additional processing would result in a measurable impact on estimates of dietary intake it was considered necessary to carry out an evaluation when collecting baseline date. The challenges experienced for ASCOT dietary assessment are likely to be encountered by others evaluating adherence to WCRF guidelines and are therefore shared here.

The aims of this study were to (i) develop methodology to process 24-h dietary recalls obtained using myfood24 to facilitate estimation of adherence to five WCRF dietary guidelines and (ii) assess the impact of performing additional data processing on estimates of dietary intake.

## Materials and Methods

### Participants and Data Collection

Participants were adults aged 18 years and older taking part in ASCOT. Details of the trial can be found elsewhere (17). In brief, participants were recruited from ten hospital sites in London and Essex *via* a survey. Hospital sites were asked to send a questionnaire to patients diagnosed with breast, prostate, or colorectal cancer between 2012 and 2015. As hospitals did not always accurately identify patients, some participants were diagnosed outside of these dates. Respondents interested in learning more about a trial of a lifestyle intervention who met eligibility criteria were asked to provide informed consent and complete additional measures of their diet and physical activity. Ethical approval was obtained through the National Research Ethics Service Committee South Central—Oxford B (reference number 14/SC/1369).

Participants were asked to complete two 24-h dietary recalls at four timepoints: baseline, 3 months, 6 months, and 2 years post-intervention. The myfood24 software was chosen, as it was the first online 24-h recall tool developed and tested with UK adults (18). It allows participants to self-administer 24-h recalls *via* the Internet as it enables participants to search for items and estimate portion sizes using pictures or standard household measures or weight (18).

On the day of the recall, participants were sent an email with a personalized link to the myfood24 website. Researchers completed recalls over the phone if a participant did not use email or struggled with online completion (19,20). Recalls completed online by participants were not routinely reviewed but if participants reported difficulties or researchers noticed unusual entries when downloading data, participants were contacted to resolve queries (19).

### Assessing Adherence to WCRF Guidance

Adherence to dietary recommendations was assessed directly using WCRF criteria where possible (e.g. ≤500 g red meat/week) or national guidelines when WCRF guidance was not quantitative (e.g. reduce intake of energy-dense foods) (Table 1).

**Table 1.** Operationalization of World Cancer Research Fund recommendations for ASCOT.

WCRF recommendation (21)	Criteria for assessing adherence to recommendation
Limit consumption of energy-dense foods and fast foods and avoid sugary drinks.	Fat intake: 33% of energy intake or less (6). Sugar intake: non-milk extrinsic sugars (NMES) make up 10% of energy intake or less (6) or free sugars makes up 5% of energy intake or less^1^ (5).
Eat at least five portions of fruit and non-starchy vegetables a day.	Fruit and vegetables (F&V): 5 or more portions (400g or more) a day (21).
Eat relatively unprocessed grains and/or pulses at each meal.	Fiber (as a measure of unprocessed grains and pulses): at least 18g non-starch polysaccharides (NSP) per day (6) or 30g AOAC fiber or more per day^1^ (3).
Limit intake of red meat.	Red meat: 500g per week or less (21) (71g per day or less).
Avoid processed meat.	Processed meat: 0g per day (21).

Abbreviation: g: grams.

^1^The national recommendations for sugar and fiber intake changed during the study so participants were assessed against both recommendations, therefore both values had to be obtained from the data.

As data collection began in 2015, recommendations were taken from the 2007 WCRF report which was available at the time (21). This included assessing intake of non-milk extrinsic sugars (NMES), which are defined as sugars that are not incorporated into the cellular structure of foods, including those added to foods or that naturally occur, for example in fruit juice (5,6). Naturally occurring sugars in milk and milk products, despite being extrinsic, are not included (5,6). As alcohol intake is often irregularly consumed, intake was assessed *via* a questionnaire rather than using the 24-h recall so is not reported here (17,21).

### Development of Dietary Quality Control Checks

Quality control checks involved identifying extreme outliers for portion sizes and daily energy and nutrient intake to identify possible errors in data entry or the nutrient database. The nutrients selected for checks were those needed to assess compliance with WCRF guidance. Changes to foods or portion sizes were made where two researchers with expertise in nutrition or dietetics agreed that it was implausible or extremely unlikely that the entry was a true reflection of consumption. Whilst alcohol intake was not assessed *via* the recalls, total alcohol intake was checked because portion size errors for alcoholic drinks could potentially impact other nutrients such as energy intake. Cutoffs for checks were developed pragmatically when exploring baseline data to ensure major errors were identified whilst keeping the number of recalls requiring a check manageable for researchers.

### Assessment of the Impact of Additional Nutrient Data Processing

Data from the first recall collected at baseline were used to assess the impact of additional data processing on estimates of dietary intake. This allowed the team to assess whether additional processing was necessary and justified. Paired sample *t*-tests were used to compare myfood24 output with data generated after additional processing.

## Results

A total of 1348 patients diagnosed with breast, prostate, or colorectal cancer were recruited to the trial between August 2015 and July 2019 and 1225 of these participants completed the first 24-h recall when requested.

### Data Processing Procedure

The final procedure developed for creating the variables required to assess adherence to WCRF guidance from myfood24 output is outlined in Table 2. The process involved using two myfood24 outputs: (i) the list of individual food items and their nutrient values; (ii) estimates of total energy and nutrient intake for the day.

**Table 2.** Data processing methodology.

Dietary component	Operationalized recommendation	Variable provided in myfood24 output	Additional variables calculated	Process for estimating intake of dietary component
Fat and total sugar	≤33% E	fat (g), E(kcal)	None	Fat (%E) = (fat*9/E)*100
	NMES ≤10% E	total sugar (g), lactose (g), E (kcal)	“Dried fruit sugar” (g) = sum of total sugar (g) in each occurance of dried fruit^a^ “F&V sugar” (g), estimated using a regression equation^b^	NMES (g) = total sugar – lactose – (dried fruit sugar*0.5) – F&V sugar then NMES (%E) = (NMES*3.75/E)*100
	Free sugar ≤5% E			Free sugar (g) = total sugar – lactose – dried fruit sugar – F&V sugar Free sugar (% E) = (free sugar*3.75/E)*100
Fruit and vegetables	≥5 portions per day. Includes fresh, canned, frozen and F&V content of composite foods. Excludes potatoes. 80g = one portion except: ≥80 g pulses and beans = one portion, ≥150 ml juice^b^ = one portion, 30g dried fruit = one portion (22)	fruit (g), vegetables (g), baked beans (g), fruit juice (ml), smoothies (ml), dried fruit (g)	“Adjusted baked beans” (g) = baked beans (g) * 0.85 (sauce). If > 80g corrected to 80g “Adjusted juice^b^” (g) = juice^b^ ≥ 150ml corrected to 80g. juice^b^ < 150 ml corrected using equation: volume/150*80 “Adjusted dried fruit” (g) = dried fruit (g)* 3	Fruit and vegetables eligible for portions (g) = fruit + vegetables + adjusted baked beans – juice^b^ + adjusted juice^b^ – dried fruit + adjusted dried fruit Portions of fruit and vegetables = fruit and vegetables eligible for portions/80
Fiber	≥18g NSP per day	NSP (g) for some food items AOAC (g) for some food items	Foods with 0g NSP: “NSP approx.” (g) = AOAC/1.3 Foods with 0g AOAC: “AOAC approx.” (g) = NSP*1.3 Fiber-containing foods showing 0g NSP and 0g AOAC replaced with similar food.	Total NSP (g) = sum of NSP values + sum of NSP approx.
	≥30g AOAC per day			Total AOAC (g) = sum of AOAC values + sum of AOAC approx
Red meat	≤71g per day	None appropriate	“Red meat approx.” (g) assigned to each item in recall^ac^	Total red meat (g) = sum of red meat approx
Processed meat	0g per day	None appropriate	“Processed meat approx.” (g) assigned to each item in recall^a,c^	Total processed meat (g) = sum of processed meat approx.

Abbreviations: E: energy; g: grams; kcal: kilocalories; NMES: non-milk extrinsic sugars; F&V: fruit and vegetables; ml: millilitres; NSP: non-starch polysaccharides.

^a^Calculated or identified manually from the list of individual items in recall.

^b^Includes fruit juices and smoothies.

^c^See methods for details.

Initial examination of baseline data identified several issues, outlined below, which prompted the decision to develop standard operating procedures to ensure processing was consistent across researchers and timepoints.

### Fat and Sugar

Fat intake (% energy) was calculated directly from myfood24 output for fat and energy assuming 9 kilocalories per gram of fat (see Table 2) (23).

Initially, to determine NMES and free sugars, food items within each recall were individually assigned values, but this was unfeasible for the large volume of data collected. Therefore, a method of approximating NMES was developed using total sugar and lactose values provided in myfood24 output. As NMES includes 50% of the sugar found in dried, stewed or canned fruit, dried fruit was estimated by summing the sugar values of each dried fruit item in a recall, and then halved (5). Stewed and canned fruit were not commonly consumed, and so the pragmatic decision was made not to adjust NMES estimates for these. Additional adjustment was necessary to subtract intrinsic sugars from fruit and vegetables. Using data from 200 recalls, sugar (g) from all fruit and vegetable items (F&V sugar) was calculated manually, and a regression equation was then derived as follows:

F&V sugar=(0.093*)(weight fruit−weight dried fruit))                   +(0.039*weight veg)

The amount of intrinsic sugar coming from other sources, for example, the sugar found in wholegrains was considered negligible and therefore not estimated. Free sugar was estimated similarly, but as the definition does not include any of the sugar in dried, stewed or canned fruit this was subtracted (see Table 2) (5). The metabolizable energy conversion factor of 3.75 kilocalories per gram was used to calculate NMES and free sugars as a percentage of energy intake (23).

### Fruit and Vegetables

Several corrections were applied to transform the values for fruit (g/day) and vegetables (g/day) in the myfood24 output to assess adherence with guidance to consume at least five portions of fruit and vegetables per day (5-a-day) (21). In the 5-a-day guidelines, one portion of vegetables is 80 g, and pulses and beans count as a maximum of one portion per day (22). As pulses and beans were not included in the myfood24 estimation of vegetables (g/day), entries of pulses and beans had to be identified manually in each recall. The most commonly eaten pulses or beans were baked beans and intakes of others were infrequent, so due to time constraints only baked beans were included in estimated vegetable intake. Each entry of baked beans was identified, adjusted to account for sauce (85% beans: 15% sauce) and portions >80 g were reduced to 80 g to align with a single portion of vegetables (see Table 2).

In 5-a-day guidance, ≥150ml of fruit juice or smoothie is considered one portion of fruit (22). Most but not all fruit juices and smoothies were included in myfood24 estimation of fruit (g/day) so each occurrence of fruit juice and smoothies in each recall was identified to enable a correction to be applied. Volumes ≥150 ml were assigned a weight of 80 g to align with a single portion of fruit, and volumes smaller than 150 ml were adjusted to correspond with the specified 80 g per portion (see Table 2), for example, 75 ml juice was converted to 40 g fruit (half a portion). Finally, all entries of dried fruit in our recalls were identified and portion weights were multiplied by three, in line with Public Health England methodology (22,24).

### Fiber

Initial analysis of baseline data, in preparation for targeted intervention delivery, identified lower than expected fiber intakes. After closer scrutiny of individual recalls, it was found that some common fiber-containing foods had been assigned a value of 0 g for AOAC fiber, NSP fiber, or both. If a value was available for either AOAC or NSP, then a conversion factor of 1.3 was applied to estimate the missing AOAC and NSP value (5). Commonly consumed foods which were known to contain fiber but where both AOAC and NSP fiber values appeared as 0 g, including oatcakes, muesli and frozen mixed vegetables, were identified in each recall and replaced with similar foods so that a fiber value would be available.

### Red and Processed Meat

Red and processed meat were not included in myfood24 output, therefore these were estimated from the food items listed in each 24-h recall. WCRF definitions of red and processed meat were used, alongside ingredient lists for composite dishes, to create mutually exclusive lists for red meat and processed meat, for example meatballs were counted as red meat and the meat component of pork pies as processed meat (25). A standard operating procedure was developed to ensure consistency when estimating meat content of composite foods and for cuts of meat recorded including bone.

### Additional Data Cleaning Required

The criteria for quality control checks on individual food items or recall totals are described in Table 3. When examining data to develop these cutoffs it was discovered that some extremely high intakes were due to errors in the myfood24 nutrient database, rather than participants selecting implausible portion sizes. For example, a commonly selected entry for cranberry juice contained 34 g sugar/100g according to myfood24 but 9.2 g sugar/100g according to label information, therefore all entries for this juice were identified and replaced with an alternative.

**Table 3.** Criteria for quality control checks.

Criteria for checks on a food entry	Criteria for checks on daily totals
Portion size of food (excluding drinks) <3 g or >400 g AOAC fiber content of food portion consumed >15 g	Energy <500 kcal or >3500 kcal Fruit and vegetables > 15 portions AOAC fiber > 40 g Free sugar > 125 g Fat > 150 g Alcohol > 80 g (10 units) Red meat > 200 g Processed meat > 150 g

Abbreviations: kcal: kilocalories; g: grams.

Portion options, including the photos presented to participants, also appeared to be responsible for some very large portions identified in the dataset. Example 1: participants selecting “fresh egg spaghetti” were shown images of cooked spaghetti, but myfood24 software treated the portion weight as uncooked and more than doubled it. Example 2: participants selecting some varieties of bacon were presented with information that 100 g was an “average portion,” the interface did not inform participants that this was four rashers, and many participants appeared to assume it was a single rasher and select three of four multiples of this, meaning 400 g portions of bacon were not uncommon in recalls. In other instances, portion options “as bought” were shown without further explanation as to whether this meant a single biscuit or a packet, or an individual or large yogurt pot. When these and similar problems were identified, entries for these items in other recalls were then checked.

It was also noticed some food descriptions may have been unclear to participants. Example 1: several recalls included large portions of concentrated orange juice, without the addition of water. Concentrated orange juice is not widely available or commonly consumed in the UK but when searching for “orange juice” in the myfood24 interface, the first results were “orange juice, freshly squeezed” followed by “orange juice, concentrated, unsweetened.” Given the tendency for participants to choose items closer to the top of lists when using recall software (26), it was considered likely that concentrated juice had been selected in place of juice reconstituted from concentrated juice, which is widely available in the UK and labeled “from concentrate” on the front of pack. Recalls including ≥100 g concentrated juice without an entry for water, were therefore identified and replaced with reconstituted juice. Example 2: an item called “Cooked sandwich ham” with portion options of 14 g and 140 g was sometimes selected as the only item consumed for lunch. Entries such as this for 140 g “Cooked sandwich ham” were replaced with a ham sandwich incorporating both slices of ham and bread.

Detailed descriptions of data processing procedures are provided in Standard Operating Procedures (https://osf.io/8wkf6/files/osfstorage/682724b92646793920c4c015, https://osf.io/8wkf6/files/osfstorage/682724bc33e47add47cea0f7, https://osf.io/8wkf6/files/osfstorage/682724b9d3384237719c4154, https://osf.io/8wkf6/files/osfstorage/682724c533e47add47cea0f9).

### Comparison of myfood24 Output with ASCOT Processed Data

Mean estimates of intake were significantly different after processing compared with original myfood24 output, see Table 4. For example, estimated fruit intake increased in 8.4% and decreased in 17.2% of recalls, with mean (SD) changes of 70.8 (47.5)g and 99.4 (129.8)g, respectively.

**Table 4.** Comparison of dietary data from myfood24 output and ASCOT dietary data after processing and cleaning (*N* = 1225).

Nutrient					Recalls where estimated intake increased after processing			Recalls where estimated intake decreased after processing		
	myfood24 output (g) Mean (SD)	Cleaned data (g) Mean (SD)	P (paired t-test)	Recalls changed N (%)	*N* (%)	increase (g) Mean (SD)	Range (g)	*N* (%)	decrease (g) Mean (SD)	Range (g)
Sugar^1^	84.05 (38.57)	44.63 (30.57)	<0.001	1225 (100)	0 (0)	0	0	1225 (100)	39.41 (21.42)	1.42–153.56
Fruit	220.61 (192.39)	209.44 (180.77)	<0.001	314 (25.6)	103 (8.41)	70.83 (47.50)	2.00–240.00	211 (17.2)	99.40 (129.78)	0.93–920.00
Vegetables	191.50 (144.39)	189.83 (143.37)	<0.001	34 (2.78)	11 (0.90)	8.02 (2.72)	1.80–12.00	23 (1.9)	92.90 (78.19)	7.13–277.00
NSP	14.17 (6.12)	14.64 (6.26)	<0.001	558 (45.56)	558 (45.6)	1.03 (1.71)	0.00–24.76	0	0	0
AOAC	8.17 (5.38)	19.64 (8.52)	<0.001	1213 (99.02)	1213 (99.02)	11.57 (6.85)	0.13–47.75	0	0	0

Abbreviations: g: grams; NSP: non-starch polysaccharides.

^1^The myfood24-generated sugar variable was compared to the estimated free sugar variable.

## Discussion

Additional data processing steps were developed to facilitate assessment of adherence to WCRF dietary guidelines using myfood24. The development and implementation of these steps was resource intensive but necessary – comparison of dietary data before and after additional processing found considerable differences. Key learnings from ASCOT demonstrate the need to closely interrogate data generated by automated recall software and highlight specific issues research teams may wish to examine when conducting dietary surveys.

Whilst it has been proposed that data collection *via* self-administered dietary recalls reduces researcher burden during data collection, additional time may be required to develop and implement methodology to process data and assess adherence to specific dietary guidelines, such as WCRF recommendations (10–13). Comparing the myfood24 output with cleaned data shows processing resulted in a mean increase or decrease of around a portion of fruit for a quarter of participants, which is a meaningful quantity when assessing intervention impact. This was primarily due to myfood24 counting fruit juice as multiple fruit portions, while dietary guidelines limit juice to one portion, regardless of quantity consumed due to its free sugar content and lack of fiber. Without additional data processing fruit intake would therefore have been overestimated for those drinking fruit juice or smoothies. Similarly, fruit intake would have been underestimated for participants consuming dried fruit, if a correction factor had not been applied to calculate the equivalent non-dried fruit weight in line with guidance (22).

There is great potential to reduce researcher burden involved in dietary data cleaning with advancements in data processing and Artificial Intelligence (AI) technologies, for example by flagging unusual values in a dataset and learning from researchers’ decisions. Much of the processing described here to generate estimates of fruit and vegetable intake that is comparable to NHS five-a-day advice could be incorporated in recall software, as is done in the Intake24 recall tool (27). A similar approach could be applied to generate red and processed meat values. Definitions for values provided would need to be included as red and processed meat definitions vary, indeed WCRF definitions potentially differ from the typical layperson understanding of these concepts. Intake24 assigns weights for beef, lamb, pork, burgers, sausages, processed red meat and other red meat to each food item, and the Automated Self-Administered 24-Hour Dietary Assessment Tool (ASA24) takes a similar approach (27,28). However, sausages and hamburgers containing preservatives are defined by WCRF as processed meat whereas sausages and hamburgers not containing preservatives are defined as red meat which adds a level of complexity to this approach (25). More precise dietary measurement would allow researchers to assess guideline adherence with greater confidence as well as facilitating a more nuanced exploration of diet-disease relationships, potentially contributing to the development of cancer-specific dietary guidance.

The absence of AOAC values in the myfood24 nutrient database at the time of our study may be explained by missing data in the UK food composition tables alongside no requirement for “back of pack” nutrient labels, which are used in myfood24, to list fiber (23,29–31). The National Diet and Nutrition Survey (NDNS) Nutrient Databank compiles AOAC values from multiple sources including recipes and estimation from NSP to ensure all foods have a value for this nutrient (32). Without additional processing of myfood24 output, 99% of participants AOAC fiber would have been underestimated by an average of 11.6 g. This change is over a third of the daily recommended amount of 30 g or more (3).

Results highlighted the importance of examining how missing data is represented in dietary analysis software and the need to explore the scope of missing data. When missing nutrient composition values are substituted with zero, it is inevitably impossible to distinguish between foods that do not contain the nutrient and foods where the value is missing. For ASCOT, addressing this issue required skilled researchers trained in nutrition to identify and correct missing fiber values but it is probable that items were overlooked, potentially leading to an underestimation of fiber intake. Teams without nutrition expertise may find this approach more challenging or may not even be aware of the issue.

Free sugars are chemically indistinguishable from total sugar and therefore must be calculated, either through an estimation based on other sugars as was done here, or on a food-by-food basis from recipes as the NDNS Nutrient Databank has done (32). This study did not have the resources required for the second approach, which would have provided greater accuracy therefore NMES and free sugars were estimated using a regression equation for sugar from fruit and vegetables. It is worth noting that as the equation was derived from data from this sample, it may not apply to other populations. As mean free sugar intake was 39.4 g/day lower than total sugar intake and there was considerable variation between participants (SD 21.4), total sugar is not an adequate proxy for free sugar (5). Given the inclusion of both AOAC fiber and free sugar in UK nutrition guidelines, the issues we encountered are likely to also impact other groups assessing compliance (5,33).

Issues identified with food names and portion sizes presented in the participant interface highlight the need for dietary analysis software to include clear and unambiguous food and portion options, both to reduce participant burden and simplify data cleaning. A problem inherent in self-administered dietary data collection is that it is impossible to ascertain what participants truly intended to report, particularly with more unusual entries but this issue is exacerbated by ambiguity in food names or portion sizes. Adding examples to food names as others have done, offering images of food packaging, and portion size pictures for the majority of foods might provide clarity, though these would need to be frequently reviewed to ensure continued accuracy (11). Indeed, nutrient databases must be regularly maintained to ensure the accuracy of food names, portion sizes, availability of items, and nutrient data, as the creators of myfood24 acknowledge (16,29). Having fewer items could allow for more efficient maintenance which has the potential to improve accuracy; indeed Intake24 reduced the number of food items in their database with this end in mind (16). Any reduction in the number of items in a database however would have to be carefully considered to ensure that its ability to reflect the diversity of diets in the UK was not adversely affected.

To reduce the need for researchers to explore recalls identified by control checks as having exceptionally large or small portion sizes, more stringent range checks could be implemented during data collection to prompt participants to review portion sizes (34). Range checks on the total values of the recall could also be performed at the point of data collection, for example highlighting to participants where the total energy intake for the day is under 500 kilocalories as Intake24 does (11) or highlighting where the energy intake is over a given amount. This would have to be balanced against the risk of increasing social approval bias and social desirability bias (35–37).

Pitfalls in using automated dietary recall systems has implications for individuals who use such systems to self-monitor their diets. It seems probable many using the software for personal monitoring would assume that the values provided are ready for use and may potentially modify their diet based on incorrect estimates.

The rigorous quality control checks employed for ASCOT uncovered a number of issues, and only those pertaining to the baseline data have been presented. Additional issues were identified in follow-up recalls which were corrected in the individual recall where identified, but not corrected throughout the dataset as it was not feasible to reprocess data collected at earlier time points. Nor was it desirable to process post-intervention data using a different methodology to that employed at baseline. The issues highlighted here, while being relevant to other myfood24 users in particular, also serve to highlight the need for vigilance and close examination of dietary data output from other software. Output data were subject to more intense scrutiny than is usual, partly due to our desire to estimate specific cancer-relevant dietary variables, rather than simply macro and micronutrient intakes. It is unclear whether our experiences are unusual as trial papers often lack a detailed description of dietary data processing methods, although these would be valuable for other research teams.

The methodology developed had several limitations. For example, the estimation of fruit and vegetables to assess compliance with 5-a-day guidance, did not account for consumption of juice at multiple time points during the day as corrections were applied at the food item level rather than participant level. Therefore, fruit intake was overestimated for the small number of participants with multiple entries for fruit juice. Further, not all pulses and beans were included in the estimation of vegetable intake. Lastly, some meat may have been incorrectly identified as red meat or processed meat, as it could not be ascertained from the data whether items such as sausages and hamburgers contained preservatives, so all sausages were categorized as processed meat, and all hamburgers as red meat.

## Conclusion

Developing methodology to assess adherence to WCRF dietary guidelines using online dietary recall software, is resource intensive but necessary. Some of this additional work could be automated by dietary data collection software or emerging AI technologies. A pragmatic approach was required to balance resources with improved accuracy of estimates of dietary intake. The additional data processing had a meaningful impact on estimates of key foods and nutrients and provided confidence that the final data was valid and appropriate for use in ASCOT.

## Data Availability

The data that support the findings of this study are available from the corresponding author, RC, upon reasonable request.
